# Pretransplant Factors and Associations with Postoperative Respiratory Failure, ICU Length of Stay, and Short-Term Survival after Liver Transplantation in a High MELD Population

**DOI:** 10.1155/2016/6787854

**Published:** 2016-11-17

**Authors:** Mark R. Pedersen, Myunghan Choi, Jeffrey A. Brink, Anil B. Seetharam

**Affiliations:** ^1^Department of Internal Medicine, Banner University Medical Center, University of Arizona College of Medicine, Phoenix, AZ, USA; ^2^Arizona State University College of Nursing and Health Care Innovation, Phoenix, AZ, USA; ^3^Arizona Transplant Associates, Phoenix, AZ, USA; ^4^Banner Transplant and Advanced Liver Disease Center, University of Arizona College of Medicine, Phoenix, AZ, USA; ^5^Division of Gastroenterology, Banner University Medical Center, University of Arizona College of Medicine, Phoenix, AZ, USA

## Abstract

Changes in distribution policies have increased median MELD at transplant with recipients requiring increasing intensive care perioperatively. We aimed to evaluate association of preoperative variables with postoperative respiratory failure (PRF)/increased intensive care unit length of stay (ICU LOS)/short-term survival in a high MELD cohort undergoing liver transplant (LT). Retrospective analysis identified cases of PRF and increased ICU LOS with recipient, donor, and surgical variables examined. Variables were entered into regression with end points of PRF and ICU LOS > 3 days. 164 recipients were examined: 41 (25.0%) experienced PRF and 74 (45.1%) prolonged ICU LOS. Significant predictors of PRF with univariate analysis: BMI > 30, pretransplant MELD, preoperative respiratory failure, LVEF < 50%, FVC < 80%, intraoperative transfusion > 6 units, warm ischemic time > 4 minutes, and cold ischemic time > 240 minutes. On multivariate analysis, only pretransplant MELD predicted PRF (OR 1.14, *p* = 0.01). Significant predictors of prolonged ICU LOS with univariate analysis are as follows: pretransplant MELD, FVC < 80%, FEV1 < 80%, deceased donor, and cold ischemic time > 240 minutes. On multivariate analysis, only pretransplant MELD predicted prolonged ICU LOS (OR 1.28, *p* < 0.001). One-year survival among cohorts with PRF and increased ICU LOS was similar to subjects without. Pretransplant MELD is a robust predictor of PRF and ICU LOS. Higher MELDs at LT are expected to increase need for ICU utilization and modify expectations for recovery in the immediate postoperative period.

## 1. Introduction

Liver transplantation (LT) is a lifesaving procedure for patients with end stage liver disease and hepatocellular carcinoma. Recent policy changes aimed at optimizing organ distribution have increased the percentage of transplants performed for recipients with Model for End Stage Liver Disease (MELD) scores greater than 35 [1, 2]. As the trend toward higher MELD scores at the time of LT continues, recipients will require increasingly advanced and intensive care as a bridge to surgery. Recent studies have suggested that increased ICU utilization and preoperative respiratory failure are not contraindications to liver transplantation, with satisfactory long-term outcomes achievable through proper patient selection [3]. However, risk factors for postoperative respiratory failure (PRF) and increased ICU utilization immediately after LT remain poorly defined.

PRF, defined as unplanned intubation or inability to extubate within 48 hours after surgery, is a common surgical complication [4]. In the general surgical population, PRF is associated with increased 30-day morality, length of stay (LOS) in the ICU, health-care expenditure, and decreased long-term survival [5–7]. In this context, risk factors predictive of PRF include type of surgery, emergency status, dependent functional status, preoperative sepsis, higher American Society of Anesthesiologists (ASA) class, acute kidney injury, and low albumin. These factors are of decreased predictive utility in the LT candidate population in which low albumin, functional dependence, and high ASA class are common; and active, uncontrolled sepsis may temporarily inactivate status on the wait list.

Previous studies identifying risk factors for PRF in liver transplant patients have had conflicting results and report experience in recipients with relatively low biologic MELD scores at the time of LT surgery, ranging from 15 to 20 [8–10]. The purpose of this investigation was to evaluate preoperative donor, host, cardiopulmonary, and surgical variables and their association with incidence of PRF and prolonged ICU LOS in a cohort of LT recipients from United Network for Organ Sharing (UNOS) Region 5.

## 2. Methods

### 2.1. Study Design

This was a retrospective analysis of 164 consecutive patients undergoing LT over a 3-year period (2011–2014) at Banner University Medical Center in Phoenix, Arizona. After institutional review board approval, inpatient and outpatient medical records were examined with clinical endpoints and variables of interest tabulated.

### 2.2. Clinical Endpoints

Primary clinical endpoints under investigation included postoperative respiratory failure and increased ICU LOS. Postoperative respiratory failure was defined as unplanned reintubation or inability to extubate within 48 hours after LT surgery. The average ICU LOS after transplant in our institution was 5.7 days with median of 3 days. Increased ICU LOS was defined as a length of stay greater than 3 days (center median). Short-term survival after transplantation was measured in days from transplantation to 1 year after transplantation.

### 2.3. Variables Predicting Postoperative Respiratory Failure and Prolonged ICU Length of Stay

Pre-LT host demographic variables included body mass index (BMI), age, smoking status, concomitant renal insufficiency, etiology of cirrhosis, pretransplant biologic MELD, and recipient cytomegalovirus (CMV) status. Pre-LT cardiopulmonary variables included LVEF, diastolic dysfunction, FEV1 < 80% predicted, FVC < 80% predicted, and FEV1/FVC < 80% predicted. Transplant surgery related variables included donor gender, warm ischemic time > 40 minutes, packed red blood cell transfusion > 6 units (cohort median), and cold ischemic time > 240 minutes.

### 2.4. Statistical Analysis

A power analysis using G*∗*Power was conducted for binary logistic regression at a 0.05 significance level, Pr(*Y* = 1∣*X* = 1) H0 of 0.2, effect size of 1.8, and a given sample size of 164 to compute achieved power. Descriptive statistics for demographic variables were performed. Baseline demographics were tabulated for the entire population for subgroups with PRF and ICU LOS > 3 days. Binary logistic regression was conducted to predict risk factors for PRF and ICU LOS > 3 days independently. Dependent variables (PRF and ICU LOS) were dichotomous. PRF was dichotomized into 0 and 1, indicating the absence and presence of PRF, respectively. ICU LOS was dichotomized into 0, indicating a LOS less than or equal to 3 days, and 1, indicating LOS greater than 3 days. We initially employed univariate regression analysis to predict potential risk factors for PRF and ICU LOS. Variables with *p* < 0.25 from the univariate logistic regression were then incorporated into multivariate logistic regression to determine final risk factors for PRF and ICU LOS.

Nagelkerke *R* squares were used for the model summary. Wald statistics were used to assess the contribution of predictors in a given model. Hosmer and Lemeshow tests were used to measure goodness-of-fit of the data in a given model. A nonsignificant chi-square value indicated a good model fit when there was little unexplained variance in the model. Survival frequencies at 1 year were compared between groups using chi-square analysis. SPSS software (Statistical Product and Services Solutions, version 22, Chicago, IL, USA) was used for statistical analyses.

## 3. Results

### 3.1. Respiratory Failure and ICU Length of Stay

A total of 164 patients were included in the study (Table 1). Of the total population, 41 (25.0%) experienced postoperative respiratory failure. Seventy-four (45.1%) patients had an ICU LOS greater than the median of 3 days and were considered to have a prolonged ICU LOS. Overall one-year survival was 90.2%. The most common indications for LT included hepatitis C with 69 patients (42.1%), followed by metabolic causes, including alcohol abuse, with 40 patients (24.4%). Fifteen (9.1%) patients had preoperative respiratory failure requiring intubation prior to transplant. Average biologic pretransplant MELD was 31.4 ± 7.3.

Baseline cardiopulmonary data was similar between PRF and the increased ICU LOS groups. No significant differences existed between hospital admission status and need for renal replacement therapy immediately prior to transplant. On echocardiograms, diastolic dysfunction (29.2%) was the most commonly seen abnormality, followed by left ventricular systolic dysfunction with an ejection fraction < 50% (1.8%). Restrictive lung patterns (frequency of FVC < 80% = 12.8%) were relatively more common than obstructive ones (frequency of FEV1/FVC < 80% = 5.5%). The incidence of FEV1 < 50% was 3 (1.8%).

### 3.2. Predictors of Prolonged Respiratory Failure

Significant predictors of postoperative respiratory failure on univariate analysis (Table 2) included BMI > 30 (OR 1.02, *p* = 0.05), pretransplant MELD (OR 1.18, *p* < 0.01), preoperative respiratory failure (OR 11.28, *p* < 0.01), LVEF < 50% (OR 6.26, *p* = 0.14), FVC < 80% (OR 2.18, *p* = 0.15), packed red blood cell transfusion > 6 units intraoperatively (OR 1.15, *p* = 0.04), warm ischemic time > 40 (OR 0.62, *p* = 0.18), and cold ischemic time > 240 (OR 3.77, *p* = 0.02). On multivariate analysis, only pretransplant MELD was found to predict PRF (OR 1.14, *p* < 0.01) after eliminating variables which showed high multicollinearity (Table 2, *R* ^2^ = 0.333; chi-square = 8.081, *p* = 0.426). The average pretransplant MELD in patients with PRF was 36.4, which was significantly higher than the group without PRF (29.8, *p* < 0.001).

### 3.3. Predictors of ICU Length of Stay

Significant predictors of an ICU LOS greater than 3 days (Table 3) on univariate analysis included pretransplant MELD (OR 1.25, *p* < 0.01), heart failure (OR 1.41, *p* = 0.06), diastolic dysfunction (OR 2.01, *p* = 0.05), FVC < 80% (OR 2.62, *p* = 0.05), FEV1 < 80% (OR 3.76, *p* < 0.01), deceased donor (OR 1.95, *p* = 0.07), and cold ischemic time > 240 (OR 2.34, *p* = 0.04). All of the patients with preoperative respiratory failure experienced an ICU LOS greater than 3 days, and the variable could not be carried forward into multivariate analysis.

On multivariate analysis, pretransplant MELD, with an OR of 1.28, was the strongest predictor of an ICU LOS greater than three days (*p* < 0.001). Restrictive PFTs defined by FVC < 80% were protective of an increased ICU LOS (OR 0.04, *p* = 0.04), except those with more severe forms of lung disease associated with a reduction of FEV1 < 80% (OR 36.88, *p* = 0.02) (*R* ^2^ = 0.516; chi-square = 5.729, *p* = 0.678). The multivariate model for increased ICU LOS was then systematically repeated with all permutations of one variable for heart failure (either all heart failure or diastolic dysfunction) and one variable for lung restriction (either FVC < 80% or FEV1 < 80%) while retaining the rest of the variables. In these models, pretransplant MELD was consistently a significant predictor of an increased ICU LOS. However, neither heart failure nor the restrictive lung disease variables were significant predictors.

### 3.4. Survival

Overall (*n* = 164) 1-year survival after orthotopic liver transplantation was 90.2% in the cohort. Although there was a trend toward lower 1-year survival in those with PRF compared to those without (81.2% versus 91.2%, *p* = 0.36) this was not significant. In those with prolonged ICU length of stay survival at 1 year was also similar at 87.5% versus 92.4% (*p* = 0.30).

## 4. Discussion

Liver transplantation, owing to its scarcity, requires unique and extensive cardiopulmonary risk stratification. Guidelines from the American Association for the Study of Liver Disease recommend cardiopulmonary risk stratification with screening for coronary artery disease, portopulmonary hypertension, and hepatopulmonary syndrome prior to liver transplantation [11]. Work-up of the aforementioned includes contrast-enhanced echocardiography and pulmonary function testing at a minimum. Patients with angiographically assessed and advanced coronary artery disease or primary lung disease may be excluded from transplantation.

PRF is a common complication after surgery and occurs after approximately 3% of surgical cases with general anesthesia [6]. Previous studies have estimated total postoperative pulmonary complications after liver transplantation to be close as high as 50% [8]. Factors such as higher incidence of pleural effusion, severe restrictive pulmonary function patterns, protein calorie malnutrition, and increased need for perioperative blood transfusions may predispose to higher rates of pulmonary complications [12]. In the general surgical population, PRF has been associated with an increased ICU LOS, 30-day mortality, and long-term mortality [4–7].

Several studies have examined predictors of pulmonary complications after liver transplant. Most of studies examine a composite of postoperative pulmonary complications, including pulmonary thromboembolism, atelectasis, pneumonia, and pleural effusion. Levesque et al. found restrictive lung patterns (OR 3.14, *p* = 0.002) and elevated INR (OR 4.95, *p* = 0.0004) to be predictive of postoperative pulmonary complications [8]. Hong et al. found elevated bilirubin (OR 2.2, *p* = 0.031) and a history of acute rejection (OR 5.4, *p* = 0.001) to be predictive of pulmonary complications within one year, though of the complications 74% (50/67) were pleural effusions seen on chest roentgenograms [10]. One study examined PRF as defined in our study, Huang et al., and identified diabetes (OR 7.55, *p* = 0.001), pretransplant respiratory failure (OR 68.85, *p* = 0.002), renal insufficiency (OR 5.93, *p* = 0.003), liver grafts from deceased donors (OR 3.44, *p* = 0.006), and use of a molecular adsorbent recirculating system (OR 14.09, *p* = 0.024) to be predictive of PRF [12].

Long-term clinical consequences of PRF and increased ICU LOS after LT continue to be explored. Some studies have found no link between PRF and increased ICU LOS and mortality. Levesque et al. found no association between PRF and mortality in patients with an average MELD of 14.8 [8]. Oberkofler et al. found no association between an increased ICU LOS and mortality in patients with an average MELD of 19.5 [9]. However, in agreement with the general surgical literature, Hong et al. found an association between PRF and mortality by Kaplan Meier analysis at one year (*p* < 0.005) though an average MELD score was not provided [10]. This was also seen by Bozbas et al., who found that a postoperative pulmonary complication (predominantly pneumonia and pleural effusion) was associated with one-year mortality, which was more pronounced in patients with a positive deep tracheal aspirate culture (*p* = 0.001) [13].

In this investigation, MELD was the only predictor of PRF and an ICU LOS > 3 days. This is consistent with previous studies, which has found various components such as high INR [8], renal failure [12], and elevated bilirubin [10] to be predictive of PRF. Our study had a markedly higher MELD score that previously reported with an average MELD of 30.0. Rates of PRF at 25% were slightly higher than previously reported (17% in Levesque, with an average MELD 14.8) [8]. Of interest, no cardiopulmonary variable was found to be predictive of PRF. Moderate to severe lung diseases causing a reduction of FEV1 were associated with an increased ICU LOS. However, the presence of restrictive lung disease itself (perhaps as a consequence of ascites) was not predictive of either PRF or an increased ICU LOS. Furthermore, the necessity for prolonged intubation before or after transplantation is not associated with decreased one-year survival.

As the average MELD for liver transplant continues to increase [1, 2], incidence of pre- and postoperative complications will continue to rise. These peritransplant complications are a consequence of increasing MELD scores and associated comorbidities such as dense portosystemic encephalopathy, pleural effusions, sarcopenia, and diuretic refractory fluid retention [14]. While complications increase the likelihood of a poor outcome, such practice is in line with the mandates of need based allocation. Numerous studies have found that select patients with preoperative respiratory failure could still achieve acceptable long-term outcomes [3, 15]. The intubated patients in this study, with an average pretransplant MELD of 32, were eligible if they had FiO_2_ ≤ 40%, positive end-expiratory pressure ≤ 10 cmH_2_O, low vasopressor requirements, and lack of an active infection. Our study, with a similarly elevated pretransplant MELD, found that preoperative respiratory failure was not predictive of postoperative respiratory failure, supporting the conclusions found in Knaak et al. These patients were, not surprisingly, at risk for an increased ICU LOS. Whether a threshold exists with regard to pretransplant MELD and survival is a subject of debate as previous investigation has found worse survival with MELD > 35 [16] while others have associated increased intensive care utilization but similar survival [17, 18].

The present study is limited by multicollinearity. Patients with pronounced left ventricular systolic dysfunction and more severe obstructive lung disease are excluded from liver transplant. In addition, these conditions share risk factors, notably age and smoking, and may coexist in up to 20% of patients [19]. It is unclear that, even with sample size several times ours, these variables would be powered enough to be significant in a multivariate logistic regression analysis.

## 5. Conclusion

Further research is needed to clarify the relationship between pre- and postoperative respiratory failure particularly in the high MELD population. With increasing average pretransplant MELD, strategies to optimize respiratory status will become more important. Importantly, increased ICU utilization may alter current patient and provider expectations for recovery in the immediate postoperative period.

## Figures and Tables

**Table 1:** Baseline demographic characteristics.

Demographic variables	Total population (*n* = 164)	PRF (*n* = 41)	ICU LOS > 3 days (*n* = 74)
Age at transplant (Years ± SD)	56.1 ± 8.7	55.3 ± 9.1	55.4 ± 8.9
Inpatient status immediately prior to transplant	50 (30.4%)	13 (31.7%)	14 (18.9%)
Gender (Male)	105 (64.0%)	24 (58.5%)	44 (59.6%)
BMI (kg/m^2^ ± SD)	28.3 ± 5.6	28.7 ± 6.3	28.4 ± 6.1
Large volume ascites	23 (14.0%)	5 (12.2%)	11 (14.9%)
Pretransplant serum albumin (g/dl)	2.3 ± 0.7	2.4 ± 0.8	2.1 ± 0.5
Pretransplant need for renal replacement therapy	65 (39.6%)	14 (34.1%)	31 (41.8%)
Pretransplant biologic MELD ± SD	31.4 ± 7.3	36.4 ± 5.8	35.8 ± 5.5
Preoperative respiratory failure	15 (9.1%)	11 (26.8%)	15 (20.3%)
Smoking history	51 (30.1%)	15 (36.6%)	21 (28.4%)
Concurrent kidney transplant	11 (6.7%)	3 (7.3%)	5 (6.7%)
Primary indication			
Hepatitis C	69 (42.1%)	10 (24.3%)	20 (27.0%)
Metabolic	27 (16.5%)	8 (19.5%)	12 (16.2%)
Any with HCC	11 (6.7%)	2 (4.9%)	8 (10.8%)
Autoimmune	40 (24.4%)	14 (34.1%)	22 (29.7%)
Nonalcoholic steatohepatitis	19 (11.6%)	8 (19.5%)	13 (17.6%)
Echocardiography			
LVEF < 50%	3 (1.8%)	2 (4.9%)	2 (2.7%)
Diastolic dysfunction	48 (29.2%)	11 (26.8%)	11 (14.9%)
Pulmonary function testing			
FEV1/FVC < 80%	9 (5.5%)	2 (4.9%)	2 (2.7%)
FVC < 80%	21 (12.8%)	7 (17.1%)	7 (9.4%)
FEV1 < 80%	23 (14.0%)	9 (21.9%)	9 (12.1%)
Donor gender (male)	105 (64.0%)	26 (63.4%)	49 (66.2%)
Donor CMV positive	104 (63.4%)	24 (58.5%)	45 (60.8%)
Warm ischemic time (minutes ± SD)	32 ± 21	28 ± 15	30 ± 15
Cold ischemic time (minutes ± SD)	198 ± 69	221 ± 79	205 ± 72
Packed red blood cell transfusion > 6 units	71 (45%)	21 (51.2%)	38 (51.3%)

PRF: postoperative respiratory failure; ICU: intensive care unit; LOS: length of stay; SD: standard deviation; BMI: body mass index; MELD: Model for End Stage Liver Disease; EF: ejection fraction; FEV1: forced expiratory volume in one second; FVC: forced vital capacity; CMV: cytomegalovirus.

**Table 2:** Univariate and multivariate analysis of PRF.

	PRF (*n* = 41)							
	Univariate				Multivariate			
Variable	*β*	OR	*p*	95% CI	*β*	OR	*p*	95% CI
Age at transplant > 65 years	−0.07	0.93	0.88	0.93–2.36				
Inpatient status prior to transplant	0.05	1.09	0.88	0.41–2.39				
BMI > 30 kg/m^2^	0.02	1.02	0.05	0.96–1.07	0.07	1.07	0.16	0.97–1.19
Large volume ascites	−0.19	0.83	0.72	0.29–2.38				
Pretransplant MELD	0.16	1.18	<0.01	1.10–1.26	0.13	1.14	<0.01	1.03–1.25
Preoperative respiratory failure	2.42	11.28	<0.01	3.37–37.89	1.96	7.06	0.06	0.97–51.57
Smoking history positive	0.38	1.46	0.32	0.69–3.07				
Kidney transplant	0.14	1.15	0.84	0.30–4.54				
Heart failure	0.03	1.03	0.90	0.69–1.52				
Diastolic dysfunction	0.04	1.04	0.92	0.47–2.31				
Left ventricular EF < 50%	1.85	6.26	0.14	0.55–71.00				
FEV1/FVC	−0.03	0.98	0.98	0.19–5.03				
FVC < 80%	0.79	2.18	0.15	0.76–6.22	−0.33	0.72	0.64	0.18–2.91
FEV1 < 80%	1.03	2.08	0.35	1.04–7.44				
Donor gender (male)	−0.02	0.98	0.95	0.47–2.04				
Deceased (versus living) donor	0.14	1.15	0.75	0.51–2.57				
Warm ischemic time > 40 minutes	−0.49	0.62	0.18	0.19–2.13	−0.06	0.94	0.93	0.22–4.03
Cold ischemic time > 240 minutes	1.33	3.77	0.02	1.35–6.37	0.82	2.28	0.26	0.55–9.40
Recipient CMV IgG	0.07	1.07	0.86	0.49–2.33				
Packed red blood cell transfusion > 6 units	0.09	1.15	0.04	0.96–1.07	0.09	1.11	0.18	0.97–1.19

PRF: postoperative respiratory failure; ICU: intensive care unit; LOS: length of stay; SD: standard deviation; BMI: body mass index; MELD: Model for End Stage Liver Disease; EF: ejection fraction; FEV1: forced expiratory volume in one second; FVC: forced vital capacity; CMV: cytomegalovirus.

**Table 3:** Univariate and multivariate analysis of ICU LOS.

	ICU LOS > 3 days (*n* = 74)							
	Univariate				Multivariate			
Variable	*β*	OR	*p*	95% CI	*β*	OR	*p*	95% CI
Age at transplant > 65 years	−0.20	0.82	0.62	0.37–1.83				
Inpatient status prior to transplant	0.65	1.95	0.04	0.100–4.04	−2.40	0.11	0.45	0.01–30.04
BMI > 30 kg/m^2^	0.01	1.01	0.77	0.95–1.07				
Large volume ascites	0.20	1.22	0.66	0.50–2.94				
Pretransplant MELD	0.22	1.25	<0.01	1.16–1.33	0.25	1.28	0.00	1.15–1.42
Preoperative respiratory failure	NA	NA	NA	NA				
Smoking history positive	−0.12	0.89	0.73	0.46–1.73				
Kidney transplant	0.03	1.03	0.97	0.30–3.51				
Heart failure	0.34	1.41	0.06	0.99–2.00	1.43	4.16	0.34	0.23–76.36
Diastolic dysfunction	0.70	2.01	0.05	0.100–4.04	−2.49	0.08	0.40	0.01–28.04
Left ventricular EF < 50%	0.91	2.49	0.46	0.22–27.98				
FEV1/FVC	0.61	1.83	0.39	0.47–7.20				
FVC < 80%	0.69	2.62	0.05	1.01–6.79	−3.31	0.04	0.04	0.01–0.86
FEV1 < 80%	1.32	3.76	<0.01	1.46–9.70	3.61	36.88	0.02	1.89–719.14
Donor gender (male)	−0.25	0.78	0.45	0.41–1.48				
Deceased (versus living) donor	0.67	1.95	0.07	0.96–3.96	0.53	1.70	0.37	0.54–5.41
Warm ischemic time > 40 minutes	−0.24	0.79	0.45	0.17–1.72				
Cold ischemic time > 240 minutes	0.85	2.34	0.04	1.08–3.60	1.69	5.39	0.07	0.85–34.12
Recipient CMV IgG	−0.16	0.85	0.64	0.44–1.66				
Packed red blood cell transfusion > 6 units	0.07	1.54	0.49	0.75–1.27				

OR: odds ratio; 95% CI: 95% confidence interval; BMI: body mass index; MELD: Model for End Stage Liver Disease; EF: ejection fraction; FEV1: forced expiratory volume in one second; FVC: forced vital capacity; CMV: cytomegalovirus.

## Abbreviations

LT: Liver transplantation
MELD: Model for End Stage Liver Disease
PRF: Postoperative respiratory failure
LOS: Length of stay.
